# The Effect of Subclinical Hypothyroidism on Coagulation and fibrinolysis: A Systematic Review and Meta-Analysis

**DOI:** 10.3389/fendo.2022.861746

**Published:** 2022-04-29

**Authors:** Qinglei Xu, Yulong Wang, Xue Shen, Yunfeng Zhang, Qingyun Fan, Wei Zhang

**Affiliations:** ^1^ Department of Endocrinology, Lanshan District Endocrinology Hospital of LinYi, Linyi, China; ^2^ Department of General Surgery, Key Laboratory of Metabolism and Gastrointestinal Tumor, Key Laboratory of Laparoscopic Technology, Shandong Medicine and Health Key Laboratory of General Surgery, The First Affiliated Hospital of Shandong First Medical University and Shandong Provincial Qianfoshan Hospital, The First Affiliated Hospital of Shandong First Medical University, Jinan, China; ^3^ Center for Pharmacovigilance, Luozhuang Market Supervisory Authority of LinYi, Linyi, China; ^4^ Department of Endocrinology, Xintai Hospital of Traditional Chinese Medicine, Tai’an, China; ^5^ Department of Endocrinology and Metabology, Shandong Key Laboratory of Rheumatic Disease and Translational Medicine, Shandong Institute of Nephrology, The First Affiliated Hospital of Shandong First Medical University and Shandong Provincial Qianfoshan Hospital, Jinan, China

**Keywords:** coagulation, fibrinolysis, subclinical hypothyroidism, cardiovascular disease, meta-analysis

## Abstract

**Background:**

Despite patients with thyroid dysfunction show obvious abnormal hemostatic indicators in the peripheral blood, the current research on whether and how subclinical hypothyroidism (SCH) influence hemostatic function (the coagulation and fibrinolytic system) still remains controversial.

**Objective:**

We conducted this study to evaluate how SCH influence on the coagulation and fibrinolytic system in human body.

**Methods:**

Prior to March 2022, Web of Science, Embase, PubMed, WanFang, CNKI data and reference lists were searched to identify eligible researches. Two of us independently extracted the data and evaluated study quality. The effect size is represented by standard mean difference (SMD). Both fixed and random-effects models were used where appropriate. Review Manager 5.3 and STATA 16.0 were used to analyze the eligible data.

**Results:**

1325 patients from twelve observational studies were involved in our research. Our study revealed that SCH changed the heamostatic balance towards hypercoagulable and hypofibrinolytic conditions accompanied by an increase in tissue fibrinogen, plasminogen activator and plasminogen activator inhibitor-1. By contrast, there was no statistically difference in acivated partial thromboplastin time (APTT) and D-Dimer in SCH group compared with that in control subjects.

**Conclusions:**

Our study confirmed that SCH is related with a prothrombotic state, as reflected by changes in both coagulation and fibrinolysis. It is highly recommended for screening cardiovascular risk factors in combination with an adequate evaluation of SCH state.

**Systematic Review Registration:**

[https://www.crd.york.ac.uk/prospero/#recordDetails] PROSPERO [CRD42021275313]

## Introduction

Subclinical hypothyroidism (SCH) is a condition associated with an elevated thyroid stimulating hormone (TSH) level with normal levels of free thyroid hormone (1). Since advances have been made recently on assays for TSH measurement with better sensitivity and specificity, SCH is becoming more prevalent, resulting in increased attention (2). Despite the fact that most patients with SCH don’t present with classical symptoms and signs of hypothyroidism due to the abnormal thyroid hormone level, the increase in atherosclerosis and cardiovascular disease in SCH patients is similar to that in patients with clinical hypothyroidism (3, 4).

Thromboembolism and cardiovascular disease are linked to various abnormalities of the haemostatic indicators related to coagulation and the fibrinolytic system. However, the published data about the heamostatic abnormalities among SCH patients remain controversial. Accumulating evidence has shown that levels of factor VII (FVII):C, the ratio FVII:C/FVII : Ag, fibrinogen (5) and plasminogen activator inhibitor-1 (PAI-1) (6, 7) are elevated, while von-Willebrand factor (vWF), antithrombin III (AT III) concentration and factor VIII (FVIII) activities (8) are decreased in patients diagnosed with SCH. However, conflicting outcomes exist in other researches (9, 10).

In this study, we aimed to analyze systematically the impact of SCH on the coagulation-fibrinolytic system in the human body, develop well-founded hypotheses, and provide recommendations for future research.

## Materials and Methods

The meta-analyses of observational researches face special challenges due to inherent biases and design differences from different studies. Hence, we conducted and detailed the analysis in accordance with the guidelines of the Meta-analysis of Observational Studies in Epidemiology Group (11).

### Search Strategy

A publication search was performed for studies in the Web of Science, PubMed, Embase, CNKI and WanFang data up to March 2022 by two independent investigators. Search strategies consisted of the following: the Medical Subject Headings terms “Hypothyroidism” or “Thyroid Disease” or “Thyrotropin”; and the text word terms “subclinical hypothyroidism” or “subclinical thyroid dysfunction” or “thyroid-stimulating hormone”; and the text word terms “haemostasis” or “blood coagulation/clotting” or “blood coagulation/clotting tests” or “blood coagulation/clotting factors” or “blood coagulation/clotting disorders”. In order to avoid omitting any relevant research, we also scanned the references of the retrieved articles for more studies. Language was not restricted in the document retrieval. Unpublished researches were not included within this study. The titles and abstracts of all retrieved articles were scanned. After that, we read the full text of studies which were possibly related for further appropriateness assessments to accomplish the article. Within the meta-analysis, all researches were firstly published in the primary literature with no reproduction in other articles. The whole researches that met the inclusion criteria were retrieved for additional assessment and information extraction.

### Inclusion Criteria

Principle consideration basis was that the research needed to assess the impact of SCH on the coagulation-fibrinolytic system in human. Take it one step further, a review need to meet the accompanying terms: 1) reported SCH whose TSH was high while free thyroxin within normal range; 2) reported the coagulation-fibrinolytic framework information (including tissue plasminogen activator (t-PA), plasminogen activator inhibitor type 1(PAI-1), fibrinogen, activated partial thromboplastin time (APTT) and D-Dimer) for SCH patients and that was contrasted with data of the control whose thyroid function was normal; and 3) the 95% CIs were given or we could compute the 95% CI with given data.

### Exclusion Criteria

The following sorts of studies were excluded: 1) Patients who were taking drugs or received treatment which could influence TSH and free thyroxin levels; 2) Participants suffered from clinical hypothyroidism or hyperthyroidism; 3) Case series, Case reports, editorials, reviews, *in vitro*, and non-human researches; 4) Because tumor may influence TSH and free thyroxin levels, researches on tumor patients were likewise removed; 5) the same study published before; 6) researches without sufficient to figure out the statistic or value.

### Study Selection and Data Extraction

Headlines and summary of these original studies were scanned to see whether the inclusion criteria were met by two researchers separately. When we could not remove a study just from headlines and summary, we needed to review this article thoroughly. Choices with respect to incorporation were made independently, outcomes were contrasted, and discussion was made for dispute resolution if there was any difference. If different publications came from the same research, we chose the recent article. When there was a need, information from other prior articles was used to replenish it. These data below was collected from every article: features of the research (writer, publication year, region, research design, exclusion criteria, TSH assay), particulars of member features (number of patients enrolled, mean age, sex and TSH level), coagulation and fibrinolysis indexes of the SCH groups and the control [fibrinogen, tissue plasminogen Activator (t-PA), PAI-1, D-Dimer and activated partial thromboplastin time (APTT)].

### Quality Assessment

The Newcastle-Ottawa Quality Assessment Scale (NOS) for evaluating quality of observational researches was utilized as a direct to evaluate research quality of cross-sectional and intervention researches (12, 13). Three categories were hence recognized: high quality (low risk of bias), medium quality (moderate risk of bias), or low quality (high risk of bias). Quality of the included researches was evaluated by two independent analysts and any contrasts were settled by agreement or the supposition of the third analyst, when necessary.

### Statistical Analysis

We drew the effect sizes that compared the experimental and non-exposure situations from each study. And then, for all eligible studies, the weighted mean difference (WMD) or Standard mean difference (SMD) and 95% CIs in coagulation and fibrinolysis indexes were calculated. Both fixed- and random-effects models were used where appropriate (14) in the meta-analysis. We examined the heterogeneity across studies through Q test and *I^2^* statistics. If *P*< 0.1 or *I^2^* ≥50%, when heterogeneity was thought to be obvious across these study outcomes, we chose the random-effect modeling the combinational analysis. If not, the fixed-effect model was used. We assessed the stability and reliability of our study through sensitivity analysis. The possible publication bias was evaluated through Egger’s and Begg’s test (15, 16). We performed the analysis by Review Manager 5.3 software (Cochrane Collaboration, http://www.cochrane.org) and STATA 16.0 software (Stata: Software for Statistics and Data Science | Stata https://www.stata.com/).

## Results

### Study Selection

**Figure 1:**
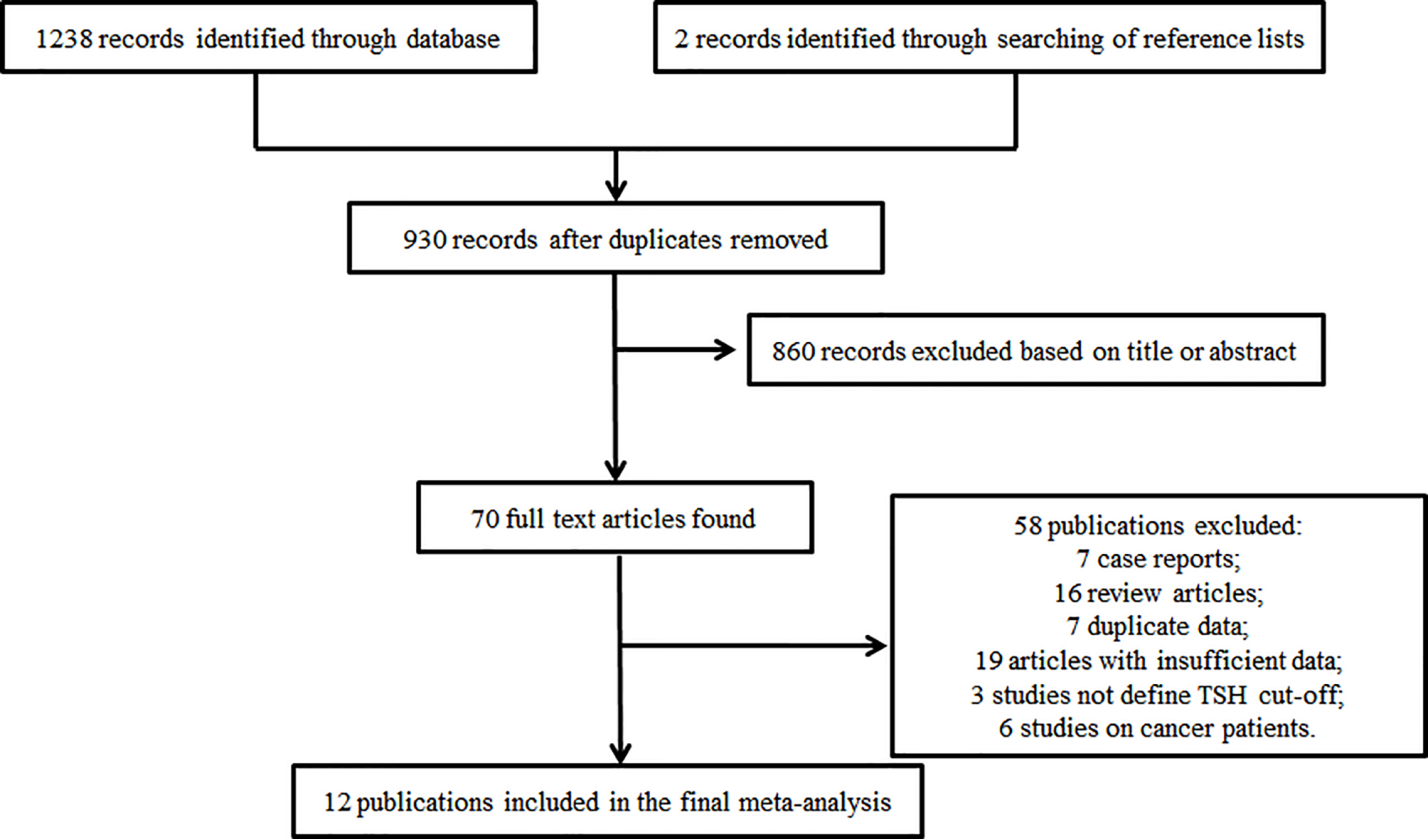
Study selection process.

**Figure 1** shows a flow diagram from which we can understand the search tactics and study selection process. The initial search tactics identified 1238 articles. Two records were involved when search of reference lists and review articles were performed further. In view of the headlines and abstract, we involved 70 potentially relevant publications in all. Of these, 58 failed to match the inclusion criteria (7 case reports,16 review articles,7 duplicate data,19 articles with insufficient data,3 studies not define TSH cut-off and 6 studies on cancer patients) and a total of 12 studies (5, 6, 8–10, 17–23) with 1325 patients were involved in the final analysis (**Figure 1**).

### Study and Patient Characteristics

**Table 1** presents the features of the individuals in the SCH and control groups and the main characteristics of these studies, including region, study design, quality, exclusion criteria, outcome parameters, TSH assay and matched or adjusted factors. Among the final 12 articles, 4 were (19, 20, 22, 23) were reported in Chinese, 8 were in English (5, 6, 8–10, 17, 18, 21). Of these, ten were case–control studies and two prospective cohort studies. Of these involved researches, four medium quality studies were confirmed. The rest of researches were confirmed to be of inferior quality. **Table 1** summed up the features of these involved researches. Moreover, **Table 2** summed up information of age, sex, the sample size and numerical value of TSH, and **Table 3** displayed the coagulation and fibrinolytic parameters.

**Table 1:** Characteristics of included studies.

First author and year of publication	Region	Study design	Quality	Exclusion criteria	Outcome parameters	TSH assay	Matched or adjusted factors
B. Müller (5)	Switzerland	Case- control study	Medium	1) male, 2) nonthyroid illnesses, 3) on medication affecting thyroid function	APTT, fibrinogen,tPA,PAI-1	By an immunoradiometric assay (h-TSH, RIA gnost, Behring).	Age and gender
Z. Cantürk (8)	Turkey	Case- control study	Low	1) received LT4 replacement therapy, 2) had previous history of external radiation, radioiodine treatment, and/or drug therapy that would cause SH, 3) with severe obesity, alcohol consumers, patients receiving drugs such as diuretics and b-blockers, 4) with diabetes mellitus, impaired glucose tolerance, coronary hearth disease, familial or secondary dyslipidemia, and hepatic, renal, or other systemic diseases	APTT, tPA, PAI-1, D-dimer, fibrinogen	By chemiluminescence immunoassay method with Immulite 2000 (DPC, Los Angeles, CA) kits	Age
M.A. Ozcan (6)	Turkey	Case- control study	Low	1) had atrial fibrillation, collagen disease, diabetes mellitus, liver or renal diseases, 2) taking any drugs effecting the levels of serum thyroid hormones	t-PA,PAI-1	By non-isotopic automated immunochemiluminometric system (ACS:180, Chiron Daignostics, UK).	Age and gender
S. Guldiken (17)	Turkey	Case- control study	Low	1) received thyroid hormone replacement therapy, 2) overt obesity (≥30kg/m^2^), 3) smoking, 4) alcohol consumption, 5) diabetes mellitus, 6) cardiac,renal,and other systemic diseases, 7) on drugs affecting haemostasis and thyroid function	D-dimer	By immunometric assay method(DPC, Immulite 2000, Los Angeles, CA)	Age and BMI
S. Gullu (10)	Turkey	Prospective cohort study	Medium	1) past or current serious medical diseases including diabetes mellitus and coronary heart disease, 2) using any medication, including aspirin or diuretics, that might affect the study parameters, 3) had symptoms and signs of clinical bleeding, 4) current smokers	APTT	By commercially available automated chemiluminescence system kits (ACS: 180, Chiron Diagnostics, East Walpole, MA, USA)	Age,BMI, gender, smoking status and blood pressure
R. Jorde (18)	Norway	Case- control study	Medium	1) a history of coronary infarction, angina pectoris or stroke in the questionnaire, 2) using thyroid medication	tPA,PAI-1	_	Age,BMI, gender,and smoking status
C. Erem (9)	Turkey	Case- control study	Low	taking drugs or had diseases (e.g.diabetes mellitus, overt obesity, coronary heart disease, collagen disease, liver cirrhosis, atrial fibrillation or renal disease) known to affect blood coagulation or fibrinolysis	APTT, D-dimer, fibrinogen, t-PA, PAI-1	By automated chemiluminescence (Bayer Corporation, Tarrytown, NY, USA)	Age and gender
Y.H. Chen (19)	China	Case- control study	Low	1) Taking estrogen, glucocorticoids, iodine, lipid-lowering drugs or β- Receptor blockers, 2) with diabetes, nephrotic syndrome, liver disease, chronic pancreatitis or familial hyperlipidemia	D-dimer,t-PA,PAI-1	ECLIA by Beckman Coulter Chemiluminescence immunoassay analyzer and kit	Age, BMI and gender
S.C. Zhong (20)	China	Case- control study	Low	TSH greater than 20 uIU/mL	APTT, fibrinogen	By automated Electrochemiluminescence immunoassay (COBAS, E411,Roche,Switzerland)	Age and gender
R. Lupoli (21)	Italy	Prospective cohort study	Medium	1) known inherited alterations in primary and/or secondary hemostasis, 2) treatment with anticoagulant or antiplatelet drugs, 3) personal and/or family history of arterial or venous thrombosis, 4) other conditions known to impact on hemostatic variables levels (liver disease, active inflammatory processes, pregnancy, malignancy, hematologic diseases, puerperium, oral contraceptive (OC) intake and hormone replacement therapy), 5) history of chronic infectious disease (including hepatitis B and C), 6) unstable medical conditions	PAI-1, t-PA,D-Dimer	By chemiluminiscent enzyme immunoassay (Elecsys E170, Roche Diagnostics, Mannheim)	Age, gender
Y.X. Ren (22)	China	Case- control study	Low	1) Hyperthyroidism and hypothyroidism, 2) Other heart diseases other than coronary heart disease, 3) Adrenal insufficiency, 4) Malignant tumor, acute cerebrovascular disease or hereditary hyperlipidemia, 5) In recent 3 months, taking drugs that affect thyroid function (such as amiodarone, thyroxine preparation, dopamine and hormone, etc.)	fibrinogen	By automated electrochemiluminescence immunoassay (COBAS8000,Roche,Switzerland)	Age, gender
F. Gao (23)	China	Case- control study	Medium	1) Age< 18 years old, 2) Taking drugs that affect thyroid function and hypolipidemic drugs, 3) with coronary heart disease, diabetes,hypertension, hyperlipidemia, chronic liver disease, chronic kidney disease, acute and chronic inflammation or connective tissue disease, 4) Postpartum or pregnancy	D-dimer, APTT, fibrinogen, t-PA, PAI-1	By automated electrochemiluminescence immunoassay (COBAS e601,Roche,Switzerland)	Age,blood pressure and BMI

APTT, activated partial thromboplastin time; t-PA, tissuetype-plasminogen activator; PAI-1, plasminogen activator inhibitor type 1; BMI, body mass index.

**Table 2:** Patient characteristics by risk factors and outcomes by trials for SCH.

First author and year of publication	TSH cutoff value	T4 measured?	Age (year)		Gender (female %)		TSH		Sample size		
			SCH	EU	SCH	EU	SCH	EU	SCH		EU
B. Müller (5)	≥6 mIU/l	Yes	59.0 ± 13.0	49.0 ± 13.0	100	100	16.0 ± 16.9	2.0 ± 1.0	42		66
Z. Cantürk (8)	–	Yes	42.2 ± 11.6	44.3 ± 6.7	100	100	8.69 ± 5.40	1.47 ± 1.04	35		30
M.A. Ozcan (6)	>5 uIU/ml	Yes	39.3 ± 13.9	46.4 ± 5.7	20.0	31.3	13.74 ± 4.85	2.09 ± 1.69	10		16
S. Guldiken (17)	>4 uIU/ml	Yes	31.0 ± 7.6	31.2 ± 6.4	100	100	7.3 ± 2.1	1.4 ± 0.8	15		15
S. Gullu (10)	>5mIU/l	Yes	47.6 (21-68)	49.2 (25-61)	100	100	7.1 (5.2-10)	1.3 (0.6-1.9)	15		15
R. Jorde (18)	>3.5 mIU/l	Yes	62.2 ± 11.8	60.8 ± 12.6	51.8	53.9	5.28 ± 1.42	1.54 ± 0.63	83		141
C. Erem (9)	>5 mIU/l	Yes	41.0 ± 13.5	41.7 ± 12.8	76.7	80	10.3 ± 5.03	1.69 ± 1.06	30		20
Y.H. Chen (19)	> 4.8 mIU/l	Yes	67.9 ± 4.8	67.8 ± 4.2	100	100	9.38 ± 2.55	–	52		50
S.C. Zhong (20)	>4.3 uIU/ml	Yes	55 (35-75)	55 (36-73)	51.7	53.3	–	–	60		30
R. Lupoli (21)	>4.5 uIU/ml	Yes	41.4 ± 13.0	42.2 ± 11.9	80.5	80.5	7.3 ± 4.8	2.1 ± 0.9	41		41
Y.X. Ren (22)	–	Yes	64.21 ± 10.38	60.92 ± 10.3	48.5	23.1	–	–	101	268	
F. Gao (23)	>4.2 uIU/ml	Yes	55.7 ± 7.78	52.11 ± 8.73	100	100	7.22 ± 3.75	1.65 ± 0.71	95	54	

**Table 3:** Coagulation and fibrinolytic changes in the two groups of each study.

First author and year of publication	APTT		D-Dimer		Fibrinogen		t-PA		PAI-1	
	SCH	EU	SCH	EU	SCH	EU	SCH	EU	SCH	EU
B. Müller (5)	26 ± 3	27 ± 3	—	—	2.5 ± 0.5	2.4 ± 0.4	6 ± 4	6 ± 4	56 ± 35	52 ± 38
Z. Cantürk (8)	29.6 ± 3.4	28.3 ± 1.8	0.52 ± 0.75	0.39 ± 0.27	329.0 ± 51.2	314.5 ± 27.1	3.60 ± 1.70	4.05 ± 1.76	19.78 ± 6.20	10.18 ± 3.61
M.A. Ozcan (6)	—	—	—	—	—	—	6.05 ± 2.61	6.27 ± 2.21	51.11 ± 20.97	46.03 ± 27.24
S. Guldiken (17)	—	—	0.29 ± 0.22	0.18 ± 0.11	—	—	—	—	—	—
S. Gullu (10)	26.9 ± 8.5	25.7 ± 7.6	—	—	—	—	—	—	—	—
R. Jorde (18)	—	—	—	—	—	—	11.1 ± 4.3	10.5 ± 5.1	15.3 ± 10.3	14.4 ± 13.1
C. Erem (9)	31.5 ± 3.1	30.0 ± 2.4	0.41 ± 0.3	0.24 ± 0.15	291.9 ± 50.5	309.6 ± 69.8	11.0 ± 8.5	8.6 ± 2.5	26.4 ± 10.2	27.1 ± 8.1
Y.H. Chen (19)	—	—	0.29 ± 0.16	0.15 ± 0.08	—	—	0.27 ± 0.16	0.42 ± 0.19	0.78 ± 0.33	0.40 ± 0.19
S.C. Zhong (20)	34.38 ± 6.56	30.41 ± 2.77	—	—	2.83 ± 0.60	2.64 ± 0.35	—	—	—	—
R. Lupoli (21)	—	—	220.3 ± 67.1	252.1 ± 72.4	—	—	5.56 ± 2.22	4.75 ± 1.61	33.6 ± 13.9	22.5 ± 5.74
Y.X. Ren (22)	—	—	—	—	3.93 ± 0.91	3.61 ± 0.89	—	—	—	—
F. Gao (23)	23.88 ± 4.46	26.19 ± 2.97	0.62 ± 0.28	0.73 ± 0.29	3.11 ± 0.69	2.67 ± 0.59	6.60 ± 2.10	5.43 ± 1.99	28.74 ± 7.35	25.77 ± 8.17

APTT, activated partial thromboplastin time; t-PA, tissue type-plasminogen activator; PAI-1, plasminogen activator inhibitor 1; —, undescribed.

### Quantitative Synthesis

#### Tissue Plasminogen Activator (tPA)

**Figure 2:**
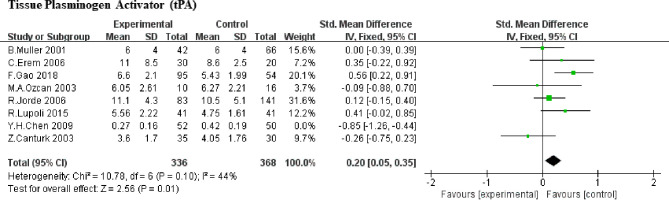
Comparison of tPA in subclinical hypothyroidism and euthyroidism.

Here, we included 8 studies (5, 6, 8, 9, 18, 19, 21, 23) (388 SCHs) for the impact of SCH on tPA. Taking the heterogeneity (heterozygosity test, Chi^2^ = 33.24, *P <*0.0001, *I^2^* = 79%) into account, 1 study was removed at a time to identify the heterogeneous source. When the research by Y. H. Chen et al. (2009) (19) was detached, the heterogeneity decreased significantly (the *I^2^* reduced from 79% to 44%, *P* increased from <0.0001 to 0.10). After a careful reading, age difference may be one of the sources of heterogeneity, but may not the only one. Therefore, SMD values was merged by means of the fixed-effect model and the pooled SMD was 0.20 (95%CI, 0.05 to 0.35; *P* = 0.01; **Figure 2**), which means that SCH patients showed higher level of tPA compared with control subjects.

#### Plasminogen Activator Inhibitor Type 1 (PAI-1)

**Figure 3:**
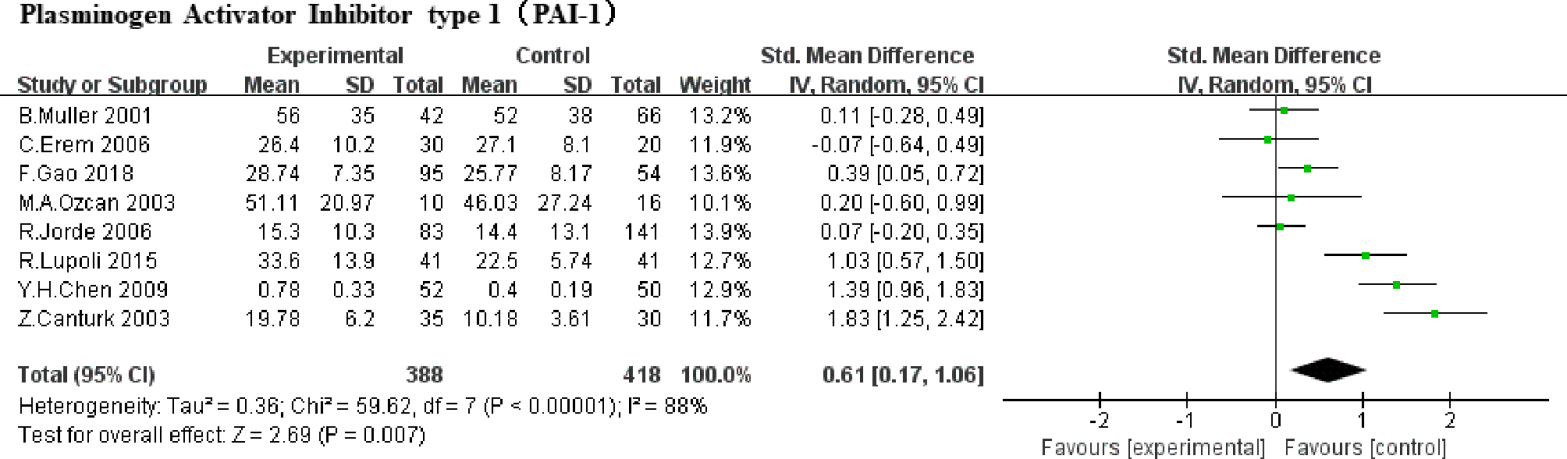
Comparison of PAI-1 in subclinical hypothyroidism and euthyroidism.

A total of 8 studies were included (5, 6, 8, 9, 18, 19, 21, 23) (388 SCHs) for the effect of SCH on PAI-1. There was significant statistical heterogeneity in these studies (*P*<0.00001, *I^2^ = *88%). Therefore, a random-effect model was used to pool SMD. A significant increase was represented when estimated together in PAI-1 among subjects in SCH group compared with the control (SMD,0.61, 95% CI 0.17 to 1.06; *P* =0.007, **Figure 3**).

#### Fibrinogen

**Figure 4:**
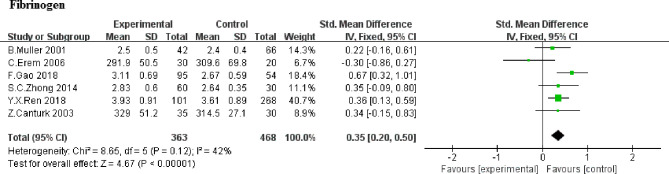
Comparison of Fibrinogen in subclinical hypothyroidism and euthyroidism.

The effect of SCH on fibrinogen was favorable in 6 studies (5, 8, 9, 20, 22, 23). Overall, the alter within the SMD for fibrinogen was 0.35 (95% CI, 0.20 to 0.50, *P*<0.00001; **Figure 4**). Heterogeneity analysis shows a moderate heterogeneity (heterozygosity test, Chi^2^ = 8.65, *P*=0.12, *I* ^2^ = 42%). It indicated that there was significantly higher in fibrinogen in SCH group, compared with euthyroid subjects. Further analysis based on whether TSH higher than 10uIU/mL or not (20, 23) showed higher fibrinogen levels in patients with TSH >10uIU/mL compared to those with TSH ≤ 10uIU/mL (WMD,0.43; 95%CI, 0.24 to 0.62; *P*<0.0001,*I^2^ = *0%). Due to the limited numbers of article, no heterogeneity was found (heterozygosity test, Chi^2^ = 0.65, *P*=0.42, *I* ^2^ = 0%).

#### Activated Partial Thromboplastin Time (APTT)

**Figure 5:**
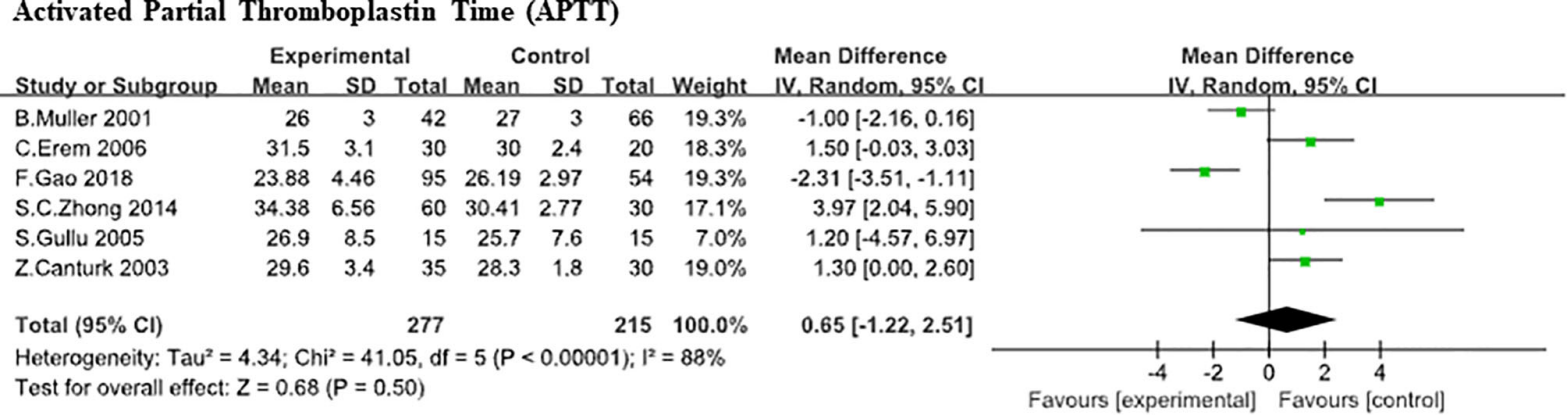
Comparison of APTT in subclinical hypothyroidism and euthyroidism.

**Figure 6:**
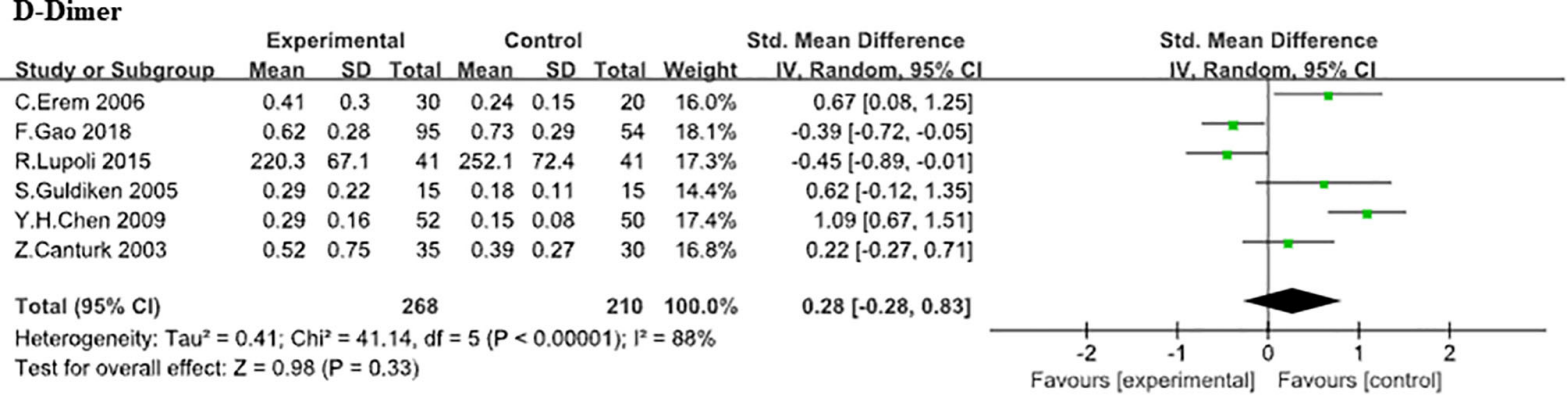
Comparison of D-Dimer in subclinical hypothyroidism and euthyroidism.

Six studies (5, 8–10, 20, 23) compared APTT levels between SCH patients with controls. Due to the large heterogeneity was found (heterozygosity test, Chi^2^ = 41.05, *P*<0.0001, *I* ^2^ = 88%), random-effect model was used to pooled the data. In our analysis, it was of no statistically difference in APTT (WMD,0.65;95% CI, −1.22 to 2.51; *P*<0.00001; **Figure 5**). Further analysis based on difference TSH level (20, 23) did not found the effect of TSH level difference on APTT level (WMD, 2.25; 95%CI, -6.86 to 11.36; *P*=0.63). Also, a large heterogeneity was found (heterozygosity test, Chi^2^ = 28.44, *P*<0.0001, *I* ^2^ = 96%), ***D-Dimer.*** Here, the association between D-Dimer and SCH was analyzed in 6 independent studies (8, 9, 17, 19, 21, 23) (268 SCHs). No statistically difference was found in D-Dimer between SCH group and normal thyroid function group (SMD, 0.28;95% CI, −0.28 to 0.83; *P* = 0.33) from analysis and the heterogeneity among trials was obvious (heterozygosity test, Chi^2^ = 41.14, *P*<0.00001, *I* ^2^ = 88%; **Figure 6**).

### Sensitivity and Subgroup Analysis

**Figure 7:**
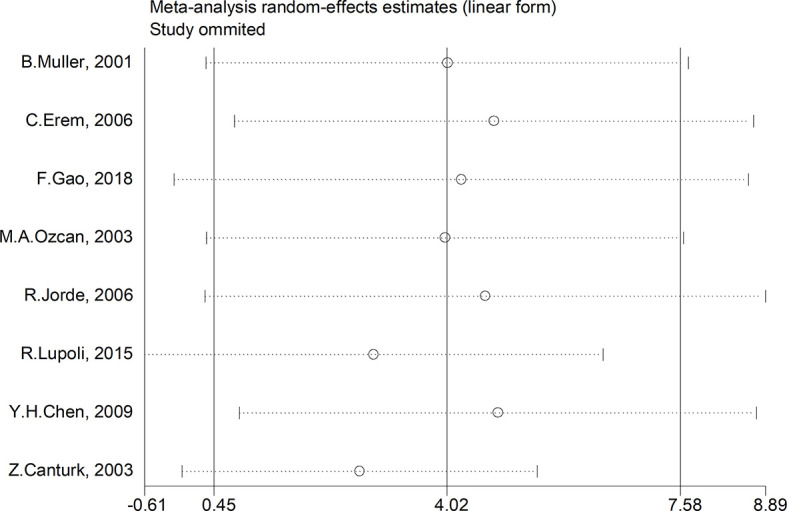
Sensitivity analysis to assess the impact of every study on the overall conclusions.

We made further efforts to conduct a subgroup and sensitivity analysis on account of study heterogeneity. Subgroup analyses were conducted by ethnicity, age, gender, TSH cut-off value and study design. However, the results of PAI-1 in SCH patients were not affected. A sensitivity analysis was performed to assess the influence of each research on the final results. There was no any single study that had an impact on the total pooled effect, sensitivity analysis, which indicated that none of the studies interfered with OR or 95% CI (**Figure 7**).

### Publication Bias Evaluation

**Figure 8:**
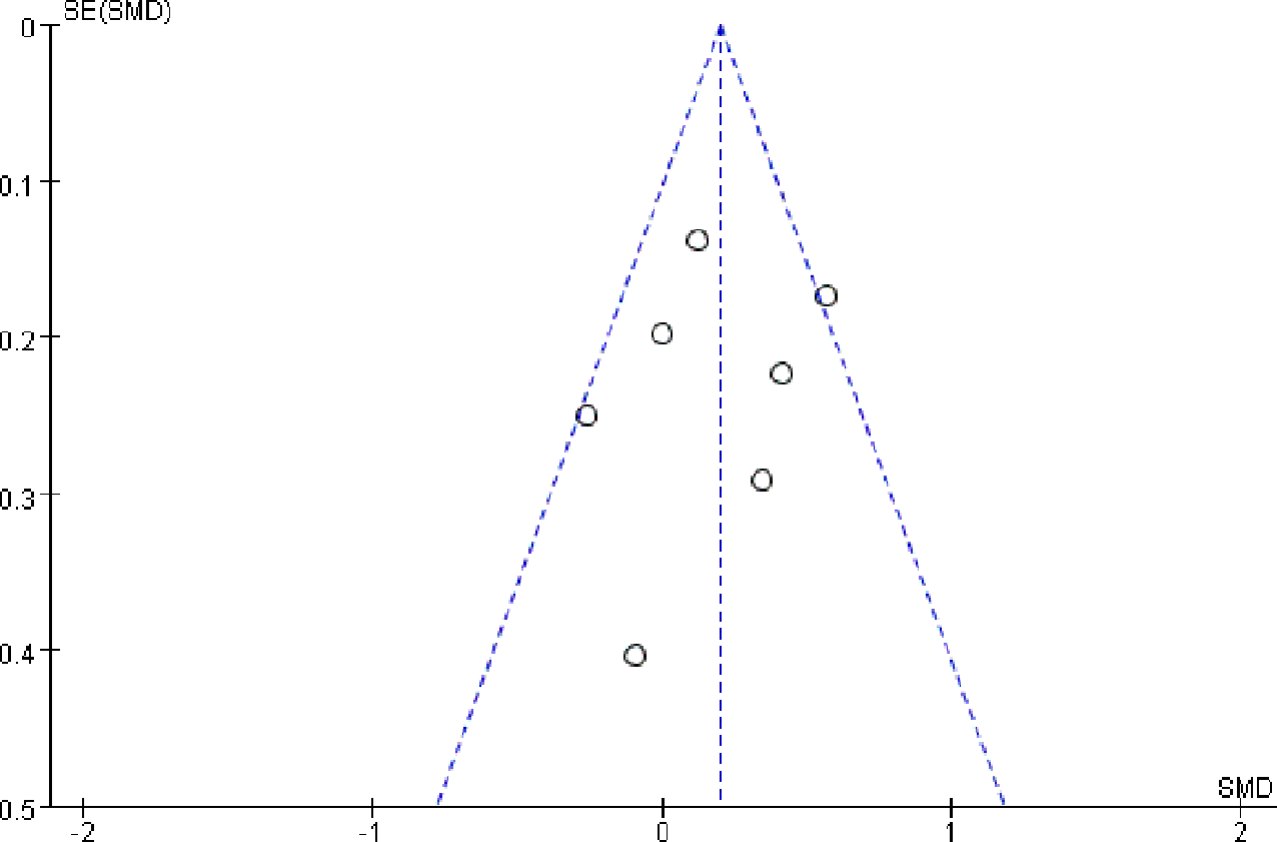
Funnel plot of publication bias-qualitative evaluation of publication bias, performed by Review Manager 5.3.

Publication bias was examined using funnel plot. There was no notable publication bias among articles included in our meta-analysis (**Figure 8**). Furthermore, no significant bias were found both by Egger’s and Begg’s test (both *P*>0.1).

## Discussion

Subclinical hypothyroidism (SCH) is defined when serum TSH is above the reference range but circulating thyroid hormones are still normal. In Iniodine-sufficient populations, SCH affects up to 16% of the population (24). Notably, researches have indicated that the risk of atherosclerosis and myocardial infarction increased independently in patients with SCH (4). However, it is still controversial that whether SCH influences coagulation and fibrinolysis in the human body. To reply this question, we did this systematic review and discovered that people suffered from SCH had hemostasis and fibrinolysis changes, consistently reflecting a prothrombotic condition.

Our study revealed that patients diagnosed with SCH displayed a prothrombotic tendency. It was showed that both t-PA and PAI-1 were above the normal range in these people, compared with the control, which indicated a hypercoagulable condition with a decline in fibrinolysis because of the pattern of alterations (21, 25). Especially, the equilibriums of t-PA and PAI-1 determine the total fibrinolytic potential of human blood, which has been extensively regarded as a predictive factor of venous as well as arterial thrombosis (26). In addition, increased t-PA levels could give expression to a compensatory reaction to a hypofibrinolysis as a result of an increased inhibitory effect of PAI-1 (26). Moreover, elevated PAI-1 concentrations may increase the tendency to myocardial infarction (MI), which suggests clinical significance of high PAI-1 levels (21, 27).

We analyzed the changes in various other hemostatic parameters and evaluate fibrinolytic balance in the current study. Especially, the increment of fibrinogen in SCH patients comparing to normal populations, which reflected both the human inflammatory state and the tendency of thrombosis and haemorrhage, contributes to atherosclerosis as well as thrombotic complications (28, 29). Data from clinical and epidemiological studies suggested that higher serum fibrinogen level could predict the risk of both primary cardiovascular events and secondary events (29–32).

Existing literatures that show an impaired fibrinolysis and a hypercoagulable in SCH patients agree to our observations (8, 17, 21, 33). This condition may be the precipitating factors of cardiovascular disease, as a mechanism how mild thyroid failure is associated with cardiovascular disease. Just as Chadarevian et al. observed (7), there was an overall decrease of fibrinolytic activity, manifested as lower D-Dimer levels, increased α2-antiplasmin reaction and increased numerical value of tPA and PAI-1, in SCH groups whose TSH level lies in 10 and 50 mIU/l. Meanwhile, Muller et al. (5) reported that subjects with SCH had a significant elevation in factor VII reaction. In a group of individuals with SCH, Canturk et al. (8) suggested elevated fibrinogen, factor VII and PAI-1 levels along with decreased AT III concentrations. It came to light that the condition resulted in more severe atherosclerosis. Furthermore, Lupoli et al. (21) have seen recovery improvements in these parameters after LT4 replacement therapy, such as a significant reduction in PAI-1 and tPA. It was concluded that SCH was a state of hypercoagulable and hypofibrinolytic and this can be reverted by L-T4 treatment. Above all, we propose the following recommendations: for patients with SCH, active LT4 treatment is recommended to reduce the incidence of thrombotic events, especially for patients with a personal history of coronary heart disease, cerebrovascular disease or early family history.

As with any study, there are some limitations in our research. Firstly, by the inclusion of no randomized clinical studies, trials with only data from observational researches, and studies mainly discussed the connection between SCH and the coagulation or fibrinolysis, it is necessary to cautiously interpret the results of our analysis (34). Secondly, the inconsistency of the standard TSH cut-off value and the definition of SCH in the final included studies may add the clinical heterogeneity in our study. Finally, factors including study samples, participants’ characteristics, the method used to test the coagulation and fibrinolysis indexes, and various confounding factors included for the adjustment, may all also added the clinical heterogeneity to our analysis.

In summary, we suggest that SCH is related to a prothrombotic state in our study, which may be caused by alterations in coagulation and fibrinolysis. Therefore, it is important to screen SCH dysfunction for detecting the earliest signs of cardiovascular disease. However, larger and high-quality studies are necessary to evaluate our observations. If future studies clearly revealed the casual association of prothrombotic state with SCH leading to an elevated risk of cardiovascular disease, effective treatments, such as replacement therapy with Levothyroxine (LT4), to revert the abnormalities should be recommended routinely.

## Data Availability Statement

The original contributions presented in the study are included in the article/supplementary material. Further inquiries can be directed to the corresponding authors.

## Author Contributions

All the authors contributed to the work. WZ and QF defined the research theme. QX and YW designed the methods, analyzed the data, interpreted the results and wrote the manuscript. XS and YZ prepared tables and figures. All authors have read and agreed to the published version of the manuscript.

## Funding

This study was supported by the Natural Science Foundation under Grant No. ZR2009CQ023 and Medical Science Development Plan of Shandong Province under Grant No. 2009QZ025.

## Conflict of Interest

The authors declare that the research was conducted in the absence of any commercial or financial relationships that could be construed as a potential conflict of interest.

## Publisher’s Note

All claims expressed in this article are solely those of the authors and do not necessarily represent those of their affiliated organizations, or those of the publisher, the editors and the reviewers. Any product that may be evaluated in this article, or claim that may be made by its manufacturer, is not guaranteed or endorsed by the publisher.
